# Action Taken by the Students of the North-Western University Dental School on the Death of Dr. Menges

**Published:** 1900-06-15

**Authors:** Elmore T. Hull, Wm. A. Kaake, L. J. Schneider, Eugene Maginnis

**Affiliations:** Committee. Chicago; Committee. Chicago; Committee. Chicago; Committee. Chicago


### ACTION TAKEN BY THE STUDENTS OF THE NORTHWESTERN UNIVERSITY DENTAL SCHOOL ON THE DEATH OF DR. MENGES.

WHEREAS, God in his infinite wisdom has removed from our midst and from the work in which he was becoming so great a force, our friend and teacher, Dr. Theodore Menges; and,

WHEREAS, We, the students of Northwestern University Dental School, realizing the great loss that is sustained thereby, be it

*Resolved*, That we bow in humble submission to the will of Almighty God, and hereby express our heartfelt sympathy to the bereaved wife and sorrowing friends, and to the Faculty of Northwestern University Dental School, of which he was a valued and honored member, and be it

*Resolved*, That each one having lost a personal friend, we express our appreciation of his untiring efforts and devotion to the upbuilding of the dental profession at large, to the Northwestern University Dental School, and to the individual interests of its students, and be it further

*Resolved*, That a copy of these resolutions be presented to Mrs. Alice Menges, to the Faculty of said institution, and that copies be sent to the leading dental journals for publication.

ELMORE T. HULL,<br>

WM. A. KAAKE,<br>

L. J. SCHNEIDER,<br>

EUGENE MAGINNIS, } *Committee.*

CHICAGO, June 2, 1900.

